# Synovial fluid leukocyte apoptosis is inhibited in patients with very early rheumatoid arthritis

**DOI:** 10.1186/ar2009

**Published:** 2006-07-19

**Authors:** Karim Raza, Dagmar Scheel-Toellner, Chi-Yeung Lee, Darrell Pilling, S John Curnow, Francesco Falciani, Victor Trevino, Kanta Kumar, Lakhvir K Assi, Janet M Lord, Caroline Gordon, Christopher D Buckley, Mike Salmon

**Affiliations:** 1 MRC Centre for Immune Regulation, Division of Immunity and Infection, The University of Birmingham, Birmingham, UK; 2 Department of Rheumatology, City Hospital, Sandwell and West Birmingham Hospitals NHS Trust, Birmingham, UK; 3 Department of Radiology, City Hospital, Sandwell and West Birmingham Hospitals NHS Trust, Birmingham, UK; 4 Department of Biochemistry and Cell Biology, Rice University, Houston, Texas, USA; 5 School of Biosciences, The University of Birmingham, Birmingham, UK

## Abstract

Synovial leukocyte apoptosis is inhibited in established rheumatoid arthritis (RA). In contrast, high levels of leukocyte apoptosis are seen in self-limiting crystal arthritis. The phase in the development of RA at which the inhibition of leukocyte apoptosis is first apparent, and the relationship between leukocyte apoptosis in early RA and other early arthritides, has not been defined. We measured synovial fluid leukocyte apoptosis in very early arthritis and related this to clinical outcome. Synovial fluid was obtained at presentation from 81 patients with synovitis of ≤ 3 months duration. The percentages of apoptotic neutrophils and lymphocytes were assessed on cytospin preparations. Patients were assigned to diagnostic groups after 18 months follow-up. The relationship between leukocyte apoptosis and patient outcome was assessed. Patients with early RA had significantly lower levels of neutrophil apoptosis than patients who developed non-RA persistent arthritis and those with a resolving disease course. Similarly, lymphocyte apoptosis was absent in patients with early RA whereas it was seen in patients with other early arthritides. The inhibition of synovial fluid leukocyte apoptosis in the earliest clinically apparent phase of RA distinguishes this from other early arthritides. The mechanisms for this inhibition may relate to the high levels of anti-apoptotic cytokines found in the early rheumatoid joint (e.g. IL-2, IL-4, IL-15 GMCSF, GCSF). It is likely that this process contributes to an accumulation of leukocytes in the early rheumatoid lesion and is involved in the development of the microenvironment required for persistent RA.

## Introduction

Inhibition of T-cell apoptosis in the synovium of patients with established rheumatoid arthritis (RA) was first described in 1995 [1]. Subsequent work contrasted the virtually complete inhibition of T-cell apoptosis in RA with high levels of T-cell apoptosis in gout [2]. The phenotype of rheumatoid synovial T cells (Bcl-X_L _^high^, Bcl-2^low^) demonstrated that their survival was maintained by stromal mechanisms rather than by the common γ-chain cytokines, and IFN-β was identified as a key fibroblast-derived survival signal [3]. In addition to their effects on T cells, both IFN-β and synovial fluid from RA patients delay neutrophil apoptosis [4,5]. These observations led to the concept that inhibition of leukocyte apoptosis, mediated by an expanded fibroblast network in the rheumatoid joint, was an important mechanism that maintains the leukocyte infiltrate in RA and perpetuates disease [6].

We recently showed that patients with very early RA, within the first 3 months of symptom onset, have a synovial fluid cytokine profile that is distinct from those of patients with other forms of very early synovitis and of patients with established RA [7]. The synovial fluid of patients with very early RA is characterized by elevated levels of cytokines that are survival factors for T cells (IL-2, IL-4 and IL-15) and neutrophils (granulocyte-macrophage colony-stimulating factor [GM-CSF] and granulocyte colony-stimulating factor [G-CSF]). We therefore sought to determine whether synovial fluid neutrophil and lymphocyte apoptosis was inhibited in patients with very early RA compared with patients with other very early inflammatory arthritides. We found that patients with very early RA had significantly lower levels of neutrophil apoptosis than did patients who developed non-RA persistent arthritis and those with a resolving disease course. Similarly, lymphocyte apoptosis was absent in patients with early RA, whereas it was seen in patients with other early arthritides. The inhibition of synovial fluid leukocyte apoptosis in the earliest clinically apparent phase of RA distinguishes this from other early arthritides.

## Materials and methods

### Patients

Patients were recruited through the rapid access clinic for early inflammatory arthritis at City Hospital, Birmingham, UK. Ethical permission was obtained and all patients gave written informed consent. All patients had one or more swollen joints and a symptom duration of 3 months or less. Patients with evidence of previous inflammatory joint disease were excluded. No patient had commenced a disease-modifying antirheumatic drug (DMARD) before initial assessment. Joints were aspirated under either palpation or ultrasound guidance. Patients were included in the study if adequate synovial fluid was obtained by palpation or ultrasound-guided aspiration/lavage at initial assessment using a method described previously [8]. Patients were subsequently assessed at 1, 2, 3, 6, 12 and 18 months. If joint effusions were present at follow-up assessments, and if consent to a further arthrocentesis was obtained, then these effusions were aspirated. Patients were assigned to their final diagnostic groups at 18 months. Patients were classified as having RA in accordance with the 1987 American Rheumatism Association criteria [9], allowing criteria to be satisfied cumulatively. Although the 1987 American Rheumatism Association criteria have no exclusions, we excluded from the RA category patients with alternative rheumatological diagnoses explaining their inflammatory arthritis. Patients were diagnosed with reactive arthritis, psoriatic arthritis, and a number of miscellaneous conditions according to established criteria.

### Synovial fluid cytospin preparation and assessment

**Figure 1:**
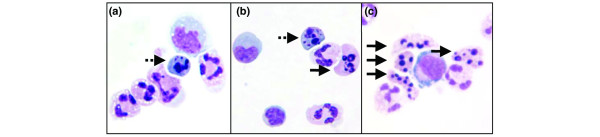
Apoptotic lymphocytes and neutrophils in synovial fluid cytospin preparations from patients with very early inflammatory arthritis. **(a) **An apoptotic lymphocyte (dashed arrow). **(b) **An apoptotic lymphocyte (dashed arrow) and apoptotic neutrophil (solid arrow). **(c) **Four apoptotic neutrophils (solid arrows).

When synovial fluid was directly aspirated from the joint, the number of leukocytes per millilitre of synovial fluid was counted using a haemocytometer; a cell count measurement was not performed when the sample was obtained by lavage. Synovial fluid cytospins were made within 30 minutes of joint aspiration or lavage [8]. The short sample processing time minimized the potential for artefactual results due to apoptosis *ex vivo*. Slides were air dried and stained with Diff-Quik (Dade Behring AG, Düdingen, Switzerland). Cytospins were assessed by an observer who was blinded to clinical details of the patients. Leukocytes were identified on the basis of morphology. Apoptotic cells were identified on the basis of a condensed or fragmented nucleus. Apoptotic neutrophils were distinguished from apoptotic lymphocytes on the basis of acidophilic or basophilic cytoplasmic staining (Figure 1). Up to 500 cells on each slide were counted where possible. A minimum of 200 cells were counted or the sample was disregarded because of lack of precision.

### Statistical analysis

Categorical variables were compared using the χ^2 ^test. Numerical variables were compared between patient groups using the Mann-Whitney test. Spearman's test was used to assess the correlation between levels of neutrophil and lymphocyte apoptosis.

## Results

### Patients

Synovial fluid was obtained at presentation from 81 patients with very early inflammatory arthritis. Characteristics of these patients at presentation are shown in Table 1. Seventeen patients developed RA. Of the 17 patients who developed a non-RA persistent inflammatory arthritis, the final diagnoses were unclassified inflammatory arthritis (*n *= 9), psoriatic arthritis (*n *= 3), arthritis related to connective tissue disease (*n *= 3), ulcerative colitis related arthritis (*n *= 1) and arthritis related to Behçet's disease (*n *= 1). Of the 47 patients whose disease resolved, the final diagnoses were unclassified inflammatory arthritis (*n *= 25), gout (*n *= 9), pseudogout (*n *= 3), reactive arthritis (*n *= 7), sarcoidosis (*n *= 1), ulcerative colitis related arthritis (*n *= 1) and psoriatic arthritis (*n *= 1). One of the patients labelled as having an unclassified resolving inflammatory arthritis fulfilled classification criteria for RA at presentation. This patient received an intramuscular injection of steroid after 10 weeks of symptoms, following which the patient remained in remission off therapy throughout follow-up. Patients whose disease progressed to RA were significantly older than patients who developed non-RA persistent synovitis or patients whose disease resolved. Patients whose disease resolved had a significantly shorter duration of symptoms at presentation compared with patients in the other two groups.

The following joints were aspirated at clinical presentation: knee (*n *= 60), ankle (*n *= 11), metacarpophalyngeal joint (*n *= 4), proximal interphalyngeal joint (*n *= 2), shoulder joint (*n *= 1), elbow joint (*n *= 1), wrist (*n *= 1) and talonavicular joint (*n *= 1). The knee was aspirated in 10 (59%) of the patients who developed RA, 15 (83%) of the patients who developed a non-RA persistent arthritis and 36 (76%) of the patients with resolving disease. There was no significant difference between the groups in terms of the numbers of patients who had the knee or another joint aspirated (χ^2 ^test; *P *= 0.22).

### Synovial fluid leukocyte apoptosis at clinical presentation

**Figure 2:**
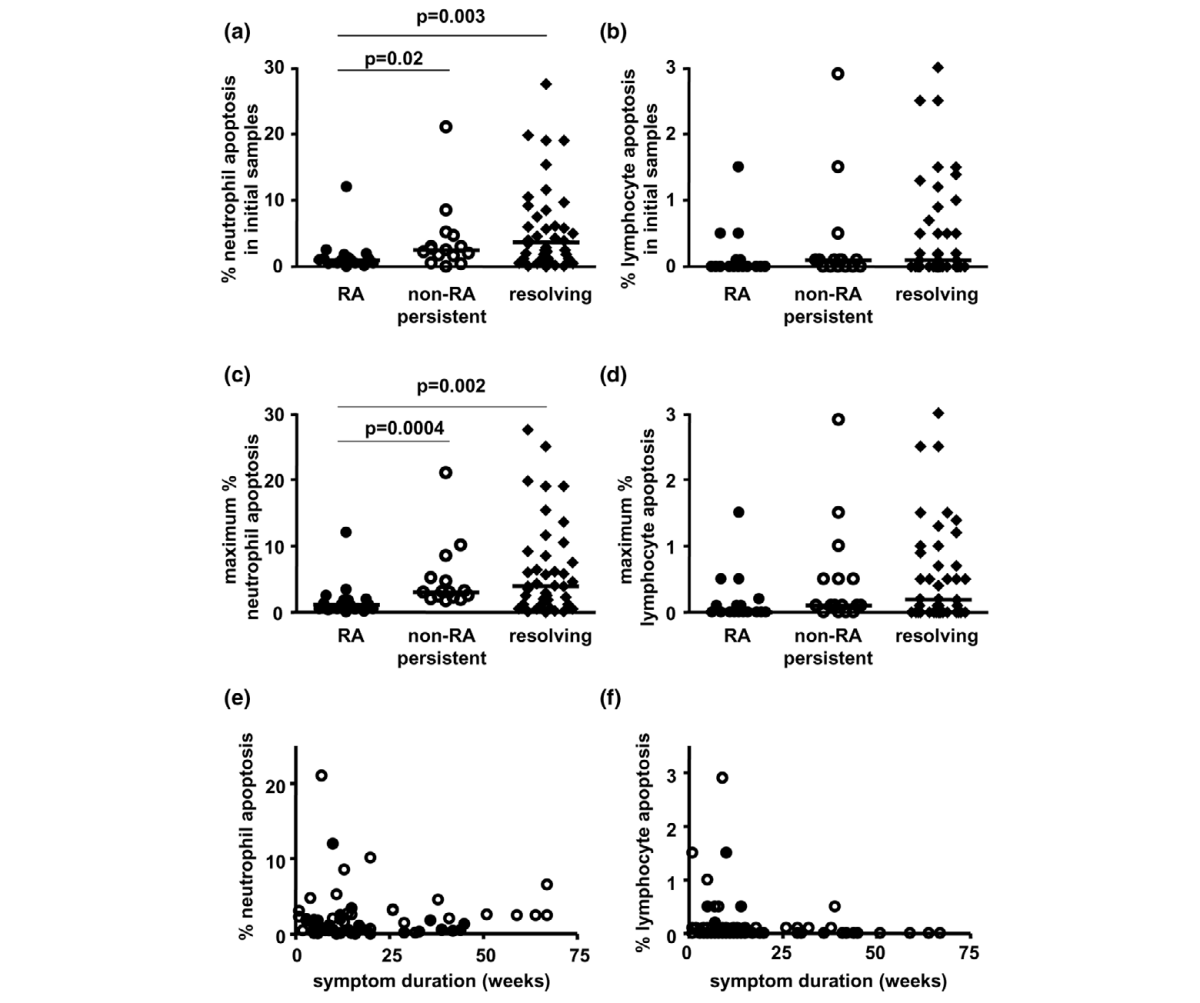
Synovial fluid neutrophil and lymphocyte apoptosis in patients with very early inflammatory arthritis. **(a) **Synovial fluid neutrophil apoptosis at clinical presentation in patients with very early inflammatory arthritis divided according to outcome. **(b) **Synovial fluid lymphocyte apoptosis at clinical presentation in patients with very early inflammatory arthritis divided according to outcome. **(c) **Maximum synovial fluid neutrophil apoptosis observed in each patient with very early inflammatory arthritis; patients divided according to outcome. **(d) **Maximum synovial fluid lymphocyte apoptosis in each patient with very early inflammatory arthritis; patients divided according to outcome. **(e) **Synovial fluid neutrophil apoptosis over time in all samples obtained from patients with very early inflammatory arthritis that eventually persisted divided according to outcome (open circles = non-RA persistent synovitis; closed circles = RA). **(f) **Synovial fluid lymphocyte apoptosis over time in all samples obtained from patients with very early inflammatory arthritis that eventually persisted divided according to outcome (open circles = non-RA persistent synovitis; closed circles = RA). Comparisons were made using the Mann-Whitney test. RA, rheumatoid arthritis.

Levels of synovial fluid neutrophil apoptosis were available for 71 patients at their initial visit. For the remaining 10 patients the number of neutrophils was too low to quantify the percentage of cells that were apoptotic. Sixteen of the 71 patients developed RA, 15 developed non-RA persistent synovitis and 40 had resolving disease. There was a significant difference in the percentage of apoptotic synovial fluid neutrophils between patients who developed RA and patients in the other two groups (RA versus non-RA persistent synovitis, *P *= 0.02; RA versus resolving synovitis, *P *= 0.003; Figure 2a). The median numbers of neutrophils per millilitre of synovial fluid in the initial samples of patients in the three groups were as follows: 2.5 × 10^6 ^(interquartile range [IQR] 0.2–6.0 × 10^6^) for patients with RA; 7.1 × 10^6 ^(IQR 3.1–22.6 × 10^6^) for patients with non-RA persistent synovitis; 3.9 × 10^6 ^(IQR 0.04–11.4 × 10^6^) for patients with resolving synovitis. Patients with RA had a lower absolute number of apoptotic neutrophils per millilitre in their initial synovial fluid samples (0.02 × 10^6 ^[IQR 0.003-0.09 × 10^6^]) than did patients with non-RA persistent synovitis (0.14 × 10^6 ^[IQR 0.02–0.99 × 10^6^]; *P *= 0.046) or patients with resolving synovitis (0.14 × 10^6 ^[IQR 0.02–0.54 × 10^6^]; *P *= 0.023).

Levels of synovial fluid lymphocyte apoptosis were available from synovial fluid cytospin preparations of 75 patients at their initial visit. For the remaining six patients the number of lymphocytes in the synovial fluid sample was too low to quantify the percentage of cells that were apoptotic. Sixteen of these patients developed RA, 15 developed non-RA persistent synovitis and 44 had resolving disease. There was a trend toward a lower level of lymphocyte apoptosis in the initial samples of patients with very early synovitis that developed into RA compared with initial samples of patients whose disease resolved, but this did not achieve statistical significance (Figure 2b). The median numbers of lymphocytes per millilitre of synovial fluid in the initial samples of patients in the three groups were as follows: 0.9 × 10^6 ^(IQR 0.1–1.4 × 10^6^) for patients with RA; 1.1 × 10^6 ^(IQR 0.8–5.0 × 10^6^) for patients with non-RA-persistent synovitis; and 0.5 × 10^6 ^(IQR 0.1–1.6 × 10^6^) for patients with resolving synovitis. There was no significant difference between the absolute numbers of apoptotic lymphocytes per millilitre in the initial synovial fluid samples from patients in the three groups.

Only an occasional apoptotic macrophage was seen, and there were no differences between groups in terms of apoptotic macrophages.

### Longitudinal assessment of synovial fluid leukocyte apoptosis

Several patients underwent repeat joint aspiration during their follow-up. Results for synovial neutrophil apoptosis from follow-up joint aspirations were available for 16 synovial fluid samples from 10 RA patients, 25 samples from 11 of the patients with non-RA persistent synovitis, and 17 samples from 11 of the patients with resolving synovitis. The number of weeks after symptom onset at which these follow-up samples were collected is shown in Table 2. More neutrophil apoptosis was seen in a follow-up sample than at initial presentation in two of the 10 RA patients, six of the 11 patients with non-RA persistent synovitis and four of the 11 patients with resolving synovitis. Prior therapy with a DMARD or parenteral steroid was not associated with enhanced levels of synovial neutrophil apoptosis at subsequent follow up (Table 2). The maximum percentage of synovial neutrophil apoptosis observed for each patient is shown in Figure 2c. The maximum level of synovial fluid neutrophil apoptosis was significantly lower in patients with RA than in patients with non-RA persistent synovitis (*P *= 0.0004) or in patients with resolving synovitis (*P *= 0.002).

Results for synovial lymphocyte apoptosis from follow-up joint aspirations were available for 12 synovial fluid samples from seven RA patients, 26 samples from 11 of the patients with non-RA persistent synovitis, and 21 samples from 13 of the patients with resolving synovitis. The number of weeks after symptom onset at which these follow-up samples were collected is shown in Table 2. More lymphocyte apoptosis was seen in a follow-up sample than at initial presentation in two of the seven RA patients, four of the 11 patients with non-RA persistent synovitis, and seven of the 13 patients with resolving synovitis. Prior therapy with a DMARD or parenteral steroid was not associated with enhanced levels of synovial lymphocyte apoptosis at subsequent follow-up (Table 2). The maximum percentage of synovial lymphocyte apoptosis observed for each patient is shown in Figure 2d. There was a trend toward patients with RA having less synovial lymphocyte apoptosis than patients with non-RA persistent synovitis (*P *= 0.09) or patients with resolving synovitis (*P *= 0.054).

Levels of synovial fluid neutrophil and lymphocyte apoptosis in initial and subsequent samples in patients who developed either RA or non-RA persistent inflammatory arthritis are shown in Figure 2e and 2f. The highest levels of leukocyte apoptosis in patients who developed non-RA persistent inflammatory arthritis were seen within the first 20 weeks of symptom onset. Only one patient who developed RA had significant levels of neutrophil apoptosis (12%) and lymphocyte apoptosis (1.5%) in a synovial fluid sample obtained after 10 weeks of symptoms. Lymphocyte and neutrophil apoptosis were virtually absent from all other synovial fluid samples from RA patients, irrespective of disease duration.

There was a statistically significant correlation between the levels of neutrophil and lymphocyte apoptosis in the synovial fluid samples (data not shown; Spearman *r *= 0.26; *P *= 0.004).

## Discussion

The early phase of RA, within 3 months of symptom onset, was characterized by very low levels of apoptotic leukocytes within the synovial compartment. During this phase of disease, patients with very early inflammatory arthritis who eventually developed RA had a significantly lower level of synovial fluid neutrophil apoptosis than did patients whose disease resolved. In addition, synovial fluid lymphocyte apoptosis was rarely observed in very early RA. There was a trend toward a higher level of lymphocyte apoptosis in patients with resolving disease compared with those who had very early RA. The inhibition of synovial fluid leukocyte apoptosis that characterizes established RA is thus also apparent in the earliest clinically apparent phase of disease. It is likely that this process contributes to the accumulation of leukocytes in the early rheumatoid lesion and is important in the development of the microenvironment that characterizes established RA. Follow-up assessments over the first 18 months of disease revealed that leukocyte apoptosis was inhibited at all time points in patients who developed RA, despite the fact that a proportion of patients had been treated, before the follow-up arthrocentesis, with antirheumatic drugs that have been reported to induce leukocyte apoptosis [10,11].

The mechanisms underlying the inhibition of leukocyte apoptosis in patients with very early RA remain undefined. In addition to IFN-β, other mechanisms for the rescue of leukocytes from apoptosis may operate in established RA. Exposure of CD4^+ ^T cells to stromal cell-derived factor-1α(SDF-1α (produced by synovial fibroblasts)) renders T cells less susceptible to apoptosis induced by anti-CD3 stimulation [12] and to cytokine deprivation induced apoptosis [13]. In Crohn's disease and experimental colitis, a role has been suggested for IL-6 trans signaling in mediating the inhibition of apoptosis in lamina propria T cells [14]. The contributions of such mechanisms to the type 1 interferon mediated T-cell rescue that operates in the rheumatoid joint is, at present, unclear. However, it is likely that the high levels of common γ-chain cytokines (IL-2, IL-4 and IL-15) and of G-CSF and GM-CSF that we recently reported in the very early rheumatoid lesion [7] play a role in lymphocyte and neutrophil survival in this phase of disease. Although these factors are not present in the joints of patients who progress to non-RA persistent disease, synovial leukocyte apoptosis was partially inhibited in these patients. Therefore, other factors are likely to contribute to leukocyte survival in the very early phase of disease that eventually persists. Macrophage-derived IFN-α is a potent survival factor for T cells and neutrophils [3,15]. Synovial macrophages may thus contribute to the inhibition of leukocyte apoptosis that is seen in early arthritides that progress to persistence.

The low level of leukocyte apoptosis in the initial samples of some patients whose disease eventually resolved was intriguing. A study of lymphocyte apoptosis during the course of a delayed-type hypersensitivity response [16] showed that levels of lymphocyte apoptosis change as the lesion develops, being absent during the generation of the response and present during its resolution. In patients with self-limiting inflammatory lesions, the level of lymphocyte apoptosis thus appears to depend on the stage of the process at which the lesion is sampled. Our results suggest a transient inhibition of lymphocyte apoptosis at early stages of the disease process in some patients with a resolving inflammatory arthritis. Potential mechanisms for this include the transient presence of soluble survival factors such as IFN-α.

In this study patients who developed RA were older than patients with other early arthritides. It has previously been reported that neutrophils from older individuals are more susceptible to spontaneous apoptosis than are neutrophils from younger persons, and that rescue from spontaneous and Fas-induced apoptosis by cytokines such as GM-CSF and G-CSF is less effective in neutrophils from older individuals than in those from younger ones [17,18]. In addition, the rate of spontaneous lymphocyte apoptosis is enhanced in elderly individuals compared with younger individuals [19,20]. Consequently, it is unlikely that the reduced leukocyte apoptosis observed in RA patients is a feature of their greater age compared with other early arthritis patients.

## Conclusion

Synovial fluid leukocyte apoptosis is inhibited during the earliest clinically apparent phase of RA. This contrasts with other early arthritides, in which significantly higher levels of neutrophil apoptosis are seen in the early lesion. The inhibition of leukocyte apoptosis in the joints of patients with RA, within the first 12 weeks of symptoms, and the presence of apoptosis in the joints of patients whose disease resolves suggest that therapies that induce leukocyte apoptosis may be useful within the first few weeks of symptoms in patients at high risk for development of RA.

## Abbreviations

DMARD = disease-modifying antirheumatic drug; G-CSF = granulocyte colony-stimulating factor; GM-CSF = granulocyte-macrophage colony-stimulating factor; IFN = interferon; IL = interleukin; IQR = interquartile range; RA = rheumatoid arthritis; SDF-1α = stromal cell-derived factor-1α.

## Competing interests

The authors declare that they have no competing interests.

## Authors' contributions

KR participated in the design of the study, recruited and followed-up the early arthritis patients, analyzed and interpreted the data, and drafted the manuscript. DST participated in the design of the study and the writing of the manuscript. CYL participated in assessing patients and in performing ultrasound-guided joint aspirations. DP participated in the design of the study and the writing of the manuscript. SJC, VT and FF contributed to the analysis and interpretation of the data. KK participated in assessing patients with early synovitis. JML participated in the design of the study and interpretation of data. CG participated in the design of the study and interpretation of data. CB participated in the design of the study and interpretation of data. MS participated in the design of the study and interpretation of data, and was involved in drafting the manuscript. All authors have read and approved the final manuscript.

## Figures and Tables

**Table 1:** Baseline characteristics of patients with very early inflammatory arthritis

Characteristic	RA	Non-RA		*P*
		Persistent	Resolving	
*n*	17	17	47	
Female (n [%])	9 (53%)	6 (35%)	24 (51%)	NS^a^
Age, years (median [IQR])	64 (58–73)	37 (25–58)	41 (29–64)	RA versus non-RA persistent: *P *= 0.001^b^; RA versus resolving: *P *= 0.0008^b^
Symptom duration (weeks; median [IQR])	7 (5–9)	7 (2.5–11.5)	3 (1–5)	RA versus resolving *P *< 0.0001^b^
CRP (median [IQR])	32 (18–53)	62 (31–95)	32 (10–96)	NS^b^
RF positive (*n *[%])	13 (76%)	1 (6%)	5 (10.6%)	

^a^χ^2 ^test. ^b^Mann-Whitney test. CRP, C-reactive protein; IQR, interquartile range; NS, not significant; RA, rheumatoid arthritis; RF, rheumatoid factor.

**Table 2:** Details of patients with very early inflammatory arthritis from whom follow-up synovial fluid samples were obtained in which neutrophil or lymphocyte apoptosis could be quantified

	RA		Non-RA			
			
			Persistent		Resolving	
	
	Neutrophil	Lymphocyte	Neutrophil	Lymphocyte	Neutrophil	Lymphocyte
Number of patients from whom analyzable follow-up samples were available	10	7	11	11	11	13
Number of analyzable follow-up samples	16	12	25	26	17	21
Number of weeks after symptom onset when follow-up samples were collected (median [IQR])	19 (15–38)	18 (13–39)	26 (14–46)	28 (14–46)	13 (9–28)	14 (9–22)
Number of patients in whom more apoptosis was seen in a follow-up sample than in the initial sample	2	2	6	4	4	7
Treatment received prior to follow-up joint aspiration where more apoptosis was seen at follow-up						
Nil	0	1	1	0	0	1
Parenteral steroid	0	0	1	1	4	6
DMARD	0	0	2	1	0	0
Parenteral steroid + DMARD	2	1	2	2	0	0
Treatment received prior to follow-up joint aspiration where no more apoptosis was seen at follow-up						
Nil	2	0	0	0	3	2
Parenteral steroid	1	0	4	6	4	4
DMARD	0	0	0	0	0	0
Parenteral steroid + DMARD	5	5	1	1	0	0

DMARD, disease-modifying antirheumatic drug; IQR, interquartile range; RA, rheumatoid arthritis.
